# Defining Skin Fibroblastic Cell Types Beyond CD90

**DOI:** 10.3389/fcell.2018.00133

**Published:** 2018-10-22

**Authors:** Dongsheng Jiang, Yuval Rinkevich

**Affiliations:** Comprehensive Pneumology Center, Institute of Lung Biology and Disease, Helmholtz Zentrum München, Munich, Germany

**Keywords:** CD90, Thy1, fibroblast, mesenchyme, fibrosis

Fibroblasts are the primary mesenchyme cell types that provide structural support during organ development and growth, and are the primary depositors of connective tissue matrix in response to injuries such as those occurring during skin scarring, tissue/organ fibrosis, systemic sclerosis, abdominal adhesions, just to name a few. Major efforts to study fibroblastic characteristics have centered on identifying surface markers that allow fibroblast purifications from tissues/organs.

The glycoprotein CD90 is a widely expressed mesenchymal cell surface marker that is the subject of almost 1,000 publications (PubMed). It is present on mesenchymal stem cells (Dominici et al., 2006), fibroblasts of various organs (Kisselbach et al., 2009) and myofibroblasts (Saada et al., 2006), and in connective tissues throughout anatomic locations, including skin (Jahoda et al., 2003; Nazari et al., 2016), liver (Katsumata et al., 2017), heart (Nural-Guvener et al., 2014), eye (Khoo et al., 2008). CD90 is also found on mesenchyme within tumors that promote tumor growth (True et al., 2010). Based on CD90s expression on various mesenchyme cell types it has been considered as a defining fibroblastic marker (Katsumata et al., 2017).

Fibroblasts were originally described as a single cell type (Ramon y Cajal, 1900). However, recent studies by us and others have demonstrated that dermal fibroblasts are an assortment of phenotypically and functionally heterogeneous cells (Driskell et al., 2013; Rinkevich et al., 2015; Singhal et al., 2016; Jiang et al., 2018; Philippeos et al., 2018). The various dermal fibroblast subtypes have drastically diverged functions, during skin development, upon wounding and at homeostasis. These different dermal fibroblast cell types can be isolated based on unique gene expression or profiles of combination of surface markers such as CD26, Blimp1, Dlk1, Sca1 in mouse (Driskell et al., 2013; Rinkevich et al., 2015), and CD26, CD39, CD36, RGS5 in human (Philippeos et al., 2018). In addition, the α5 chain of collagen VI (COL6A5) is highly enriched in human papillary fibroblasts (Fitzgerald et al., 2008), and the discoidin-domain receptor 2 (DDR2) enriches for human cardiac fibroblasts (Goldsmith et al., 2004).

CD90 alone therefore is not an accurate marker to define fibroblasts in general or its subtypes, because of the following reasons:

First, CD90 cannot distinguish dermal mesenchymal stem cells from dermal fibroblasts (Haniffa et al., 2009; Chang et al., 2014). Although CD90 is used as a mesenchymal stem cell marker (Dominici et al., 2006), it is identically expressed on the more differentiated dermal fibroblasts (Halfon et al., 2011; Fang et al., 2017).

Second, CD90 does not discriminate between functionally unique fibroblast subtypes. Fibroblasts are functionally heterogeneous, both between and within anatomic skin locations. For example, the oral cavity skin quickly seals open wounds with minimal scarring, while back-skin responds to injuries with an opaque plug of dense connective tissue. Fibroblasts taken from various skin depths also diverge functionally (Sorrell and Caplan, 2004; Philippeos et al., 2018). Fibroblasts from the upper skin layer (papillary) are pro-regenerative, whereas the fibroblasts from the deeper skin layer (reticular) are pro-fibrotic (Driskell et al., 2013). In addition to the anatomic heterogeneity, our group recently identified two additional different fibroblasts lineages in mouse back-skin that can be distinguished based on their transient early embryonic expression of *Engrailed-1* (*En1*) gene. The two fibroblast lineages either do or do not have a past history of *En1* expression, and are referred to as “En1-Past” Fibroblasts (EPFs) and “En1-Naive” Fibroblasts (ENFs). Using genetic lineage tracing and live imaging tools, we demonstrated that EPFs are the primary contributors to the scar phenotype in the back in all injury models tested, including cutaneous wounding and irradiation-induced tissue fibrosis, whereas ENFs do not participate or contribute to scarring (Rinkevich et al., 2015). In early fetal wounds ENFs typically sculpt and regenerate the original connective-tissue foundation (Jiang et al., 2018). The relative proportion of the two fibroblastic lineages changes radically during skin development, from an ENF-predominant to an EPF-predominant dermis, which leads to the skin's phenotypic transition from regeneration to scarring (Jiang et al., 2018). The flow cytometric analysis of CD90 surface expression on back-skin fibroblasts from embryonic day 18.5 (E18.5) mouse embryos reveals that CD90 is expressed on most, but not all, cells of both the EPF and ENF lineages (Rinkevich et al., 2015). This indicates that CD90 does not accurately mark any functional fibroblast subtype, nor does CD90 discriminate between anatomically distinct fibroblasts. For example, CD90 is expressed by fibroblasts in all layers of dermis, including papillary dermis, reticular dermis and hypodermis (Driskell et al., 2013), as well as different anatomic sites with diverged scarring and regenerative outcomes such as oral cavity and back-skin.

Thirdly, by large scale screening of surface markers we found that CD90 does not mark a single defined skin fibroblast subtype, and this was recently confirmed by single-cell transcriptome analysis, in both human and mouse (Rinkevich et al., 2015; Singhal et al., 2016; Philippeos et al., 2018). Therefore, future charting fibroblast subpopulations cannot rely on markers like CD90 but rather rely on lineage tracing studies that determine the fibroblast subsets based on distinct cellular functions.

The functional role of CD90 on the surface of fibroblasts is not well understood, with just a handful of intimations. CD90 on cancer-associated fibroblasts has been shown to contribute to inflammation by promoting fibroblast release of IL-6, which promotes tumor progression (Shiga et al., 2015; Huynh et al., 2016). On dermal fibroblasts, recent studies suggest that CD90 functions to dampen the expressions of the adipogenic markers PPARγ, and the Src-family kinase Fyn (Woeller et al., 2015), thereby blocking the differentiation of mesenchymal cells into adipocytes. However, the conclusion was derived from the experimental setup with a preadipocyte cell line under adipogenic induction. A direct lineage relationship between fibroblasts and adipocytes has not been directly proven so far. The observation that primary human and mouse adipocytes do not express CD90 (Phipps et al., 2012; Woeller et al., 2015) may simply suggest that adipocytes diverge from fibroblasts prior to and regardless of CD90 expression. Interestingly, a recent study shows that CD90 coupled with integrin regulates Src-family kinases at focal adhesions. Thus, CD90 may play a role in fibrogenesis by contributing to environmental rigidity sensing (Fiore et al., 2015). However, it is not clear whether this is a unique function of CD90 or a common property of a family of structurally related proteins that can bind integrins to trigger their conformational changes and alter their signaling function.

The relationship of CD90 to fibrosis is indeed far from clear. The loss of CD90 from lung fibroblasts is observed in idiopathic pulmonary fibrosis (Sanders et al., 2008) and results in a more severe fibrotic outcome (Hagood et al., 2005). In contrast, CD90 expression positively correlates with fibrosis on dermal fibroblasts in systemic sclerosis (Nazari et al., 2016) and on liver fibroblasts in cholestatic liver injury (Katsumata et al., 2017). The expression of matrix metalloproteinase inhibitor Timp1 is found to be upregulated in CD90^+^ fibroblasts near the portal vein, suggesting these cells inhibit collagen degradation and promote accumulation of extracellular matrix (Katsumata et al., 2017). In line with these observations, CD90 expression is elevated in the capsular contracture and scar tissue after the implant-based breast reconstruction, and CD90 expression is essential for the myofibroblast phenotype in capsular fibroblasts (Hansen et al., 2017). In addition, higher CD90 expression is found to be accompanied by the increment of α-SMA^+^ stromal component in hepatocellular carcinoma (Sukowati et al., 2013), implying for fibrotic and tumor promoting functions of CD90.

Defining fibroblast subsets based on their specific function could pave the way for clinical applications, and defining surface markers that mark specific classes of fibroblasts is a first step toward their cell enrichment. For examples, we have learned from previous studies that upper dermis fibroblasts promote the hair follicle regeneration, whereas lower dermis fibroblasts participate in wound healing (Driskell et al., 2013; Rognoni et al., 2016). Transplanted ENFs reduce skin scarring (Jiang et al., 2018). The functions of the specific fibroblast subsets are derived from animal studies, and the human homologous requires further investigation. The shrouded functions of CD90 on fibroblasts may arise from the heterogeneous compositions of fibroblasts in tissues. In which, CD90 relays multiple or opposing functions in distinct fibroblast lineages. The fibroblast lineage-specific functions of CD90 await future investigations.

We anticipate that fibroblasts exhibit a panoply of functionally distinct cell types, rivaling that of the hematopoietic system – different subsets carry different physiological functions and may display distinguishing surface markers. While the marker definition for hematopoietic subsets has been well established, the identification of markers for the various fibroblast subsets has just begun. The accumulating single cell transcriptome data on stromal cells from various tissues/organs including skin, may shed light on the unique markers for fibroblast subsets, and would pave the way toward cell enrichment strategies for clinical use.

## Author contributions

All authors listed have made a substantial, direct and intellectual contribution to the work, and approved it for publication.

### Conflict of interest statement

The authors declare that the research was conducted in the absence of any commercial or financial relationships that could be construed as a potential conflict of interest.
